# The potential of a multiplex high-throughput molecular assay for early detection of first and second line tuberculosis drug resistance mutations to improve infection control and reduce costs: a decision analytical modeling study

**DOI:** 10.1186/s12879-015-1205-4

**Published:** 2015-10-26

**Authors:** AH van’t Hoog, I. Bergval, N. Tukvadze, S. Sengstake, R. Aspindzelashvili, RM Anthony, F. Cobelens

**Affiliations:** Department of Global Health, Academic Medical Centre, University of Amsterdam, Amsterdam, The Netherlands; Amsterdam Institute for Global Health and Development, Amsterdam, The Netherlands; KIT Biomedical Research, Royal Tropical Institute, Amsterdam, The Netherlands; National Tuberculosis Reference Laboratory, National Center for Tuberculosis and Lung Diseases, Tbilisi, Georgia; KNCV Tuberculosis Foundation, The Hague, The Netherlands

## Abstract

**Background:**

Molecular resistance detection (MRD) of resistance to second-line anti-tuberculous drugs provides faster results than phenotypic tests, may shorten treatment and allow earlier separation among patients with and without second-line drug resistance.

**Methods:**

In a decision-analytical model we simulated a cohort of patients diagnosed with TB in a setting where drug resistant TB is highly prevalent and requires initial hospitalization, to explore the potential benefits of a high-throughput MRD-assay for reducing potential nosocomial transmission of highly resistant strains, and total costs for diagnosis of drug resistance, treatment and hospitalization. In the base case scenario first-line drug resistance was diagnosed with WHO-endorsed molecular tests, and second-line drug resistance with culture and phenotypic methods. Three alternative scenarios were explored, each deploying high-throughput MRD allowing either detection of second-line mutations in cultured isolates, directly on sputum, or MRD with optimized markers.

**Results:**

Compared to a base case scenario, deployment of high-throughput MRD reduced total costs by 17-21 %. The period during which nosocomial transmission may take place increased by 15 % compared to the base case if MRD had currently reported suboptimal sensitivity and required cultured isolates; increased by 7 % if direct sputum analysis were possible including in patients with smear-negative TB, and reduced by 24 % if the assay had improved markers, but was still performed on cultured isolates. Improved clinical sensitivity of the assay (additional markers) by more than 35 % would be needed to avoid compromising infection control.

**Conclusions:**

Further development of rapid second-line resistance testing should prioritize investment in optimizing markers above investments in a platform for direct analysis of sputum.

**Electronic supplementary material:**

The online version of this article (doi:10.1186/s12879-015-1205-4) contains supplementary material, which is available to authorized users.

## Background

Tuberculosis (TB) remains a major health problem. Multidrug-resistant tuberculosis (MDR-TB) is present in 3.6 % of new and 20 % of previously treated TB cases globally, and in over 20 and 50 % of TB patients respectively in some of the Eastern European and former Soviet Union countries [1]. MDR-TB is defined by resistance to at least isoniazid (INH) and rifampicin, the most powerful first-line anti-TB drugs [2]. Patients with additional resistance to second-line drugs constitute 32 % of MDR-patients globally [1]. These include patients with extensively drug resistant (XDR) TB, i.e. resistance to any fluoroquinolone (FQ) and to at least one of three second-line injectable drugs (SLID) capreomycin, kanamycin and amikacin [2] in addition to multidrug-resistance, or with resistance to one of these drug-classes (pre-XDR-TB). Phenotypic testing methods to determine drug susceptibility (DST) are reproducible and presumed to correlate with clinical response for most drugs, but take at least 3–5 weeks after initial culture and require a biosafety level-3 laboratory [3, 4]. In settings where additional resistance to second-line drugs is common, the influence of phenotypic DST on the selection of the proper initial treatment and containment of the spread of MDR-TB and (pre-)XDR-TB is therefore limited.

Molecular assays provide much more rapid results than phenotypic DST [3–5] and if results are correlated with clinical response have a number of potential benefits. Rapid knowledge of resistance mutations for first- and second-line drugs prior to initiation of anti-TB therapy would increase the probability that an effective treatment regimen is selected at treatment onset rather than a (gradual) adjustment from an empirical presumptive regimen to an individualized regimen due to the delayed knowledge of drug resistance [6]. It would also shorten the time that patients with additional resistance to second-line drugs remain infectious due to inadequate empirical treatment. Especially in countries where MDR patients are hospitalized during the first several weeks to months of treatment until sputum cultures are negative [1], earlier resistance results for second-line drugs would allow better infection control. Earlier separation of MDR-TB from pre-XDR and XDR-TB patients could help control nosocomial transmission of highly resistant strains [7]. Further, earlier initiation of individualized treatment could potentially reduce costs for drugs and patient care if it shortens the duration of the overall treatment period.

In the context of the development of a high-throughput multiplex assay for molecular resistance detection (MRD), we used a decision analysis to explore the potential benefits of obtaining second-line drug resistance information faster by a high-throughput MRD-assay that requires cultured isolates [8]. The example for our model is the Multiplex Ligation-dependent Probe Amplification (MLPA) technology which relies on amplification of sequence-specific probes rather than amplification of genetic targets and allows multiplexing of up to 50 genetic markers in the Mycobacterium tuberculosis genome [8]. A pilot demonstrated operational feasibility of a prototype of this high-throughput technology in a regional laboratory in a high MDR-TB setting. The clinical accuracy of the prototype assay in detecting molecular resistance to first and second-line drugs is under evaluation, and is influenced by the composition of genetic markers targeted [8]. We explored the potential of this high-throughput MRD technology for reducing nosocomial transmission of (pre-)XDR-TB after TB diagnosis and cost for treatment, hospitalization and diagnosis of drug resistance, assuming clinical accuracy as published for another MRD technology [9]. In addition, we explored how much these outcomes could be improved by allowing direct testing of sputum (optimized analytical sensitivity [10]) versus optimization of molecular targets to improve clinical accuracy [10].

## Methods

**Fig. 1:**
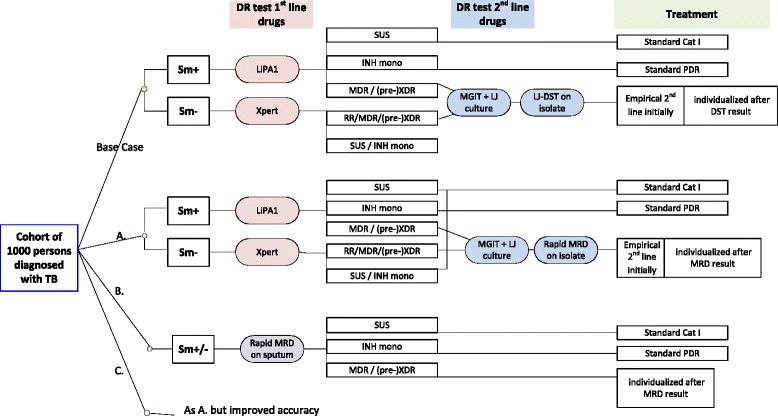
Schematic presentation of the modeled scenarios. Legend/footnote: Scenarios: **a**. = Rapid test following culture; **b**. Improved analytical sensitivity; **c**. Improved clinical accuracy; TB = pulmonary tuberculosis; DR = drug resistance; Sm + =sputum smear positive; Sm- = sputum smear negative; LiPA1 = Line Probe Assay for first-line drugs; Xpert = Xpert MTB/RIF assay; MRD = Molecular resistance detection; MGIT = Mycobacterial Growth Inhibitor Tube; LJ = Löwenstein-Jensen; DST = Drug Susceptibility Testing; SUS = susceptible TB; INH mono = isoniazide mono resistance; RR = rifampicin resistance; MDR = multi-drug resistance, defined as resistance to rifampicin and isoniazid; XDR = extensively drug-resistant tuberculosis; PDR = poly drug resistance (to first-line drugs but not rifampicin)

We modeled a cohort of patients who were diagnosed with TB and simulated four scenarios, representing different diagnostic algorithms for diagnosing drug-resistant TB and different levels of optimization of the high-throughput multiplex assay for MRD (Fig. 1, Table 1). The analysis took a TB program perspective and considered costs and effects that occurred while patients were taken in care of by the TB program from the moment of TB diagnosis (since that is the time that can be altered by the test of interest). The primary outcomes were total costs for diagnosis and treatment and potential nosocomial transmission person months (PNTPM), which were infectious person-months (IPM) during which a patient could nosocomially transmit a pre-XDR or XDR *Mycobacterium tuberculosis* (MTB) strain to other TB patients during joint hospitalization. This included potential transmission of (pre-)XDR-TB to patients with MDR-TB and pre-XDR-TB to patients with pre-XDR-TB but not the same resistance pattern (i.e. either SLID or FQ). PNTPM ended when the correct drug-resistance pattern was identified and correct infection control measures could be taken. Additional outcomes were the total IPM from the moment of TB diagnosis until sputum culture conversion, number of patients requiring future retreatment, and death.

**Table 1:** Overview of the four diagnostic scenarios to diagnose drug-resistant tuberculosis that are compared in the model

		Base case	Deployment of high-throughput MRD assay		
Scenario			A. MRD assay following culture	B. Improved analytical sensitivity	C. Improved clinical accuracy
Resistance test for first-line drugs					
Assay	Smear+	LiPA1	LiPA1	rapid MRD	LiPA1
	Smear-	Xpert	Xpert	rapid MRD	Xpert
Specimen		directly on sputum	directly on sputum	directly on sputum	directly on sputum
Accuracy: sensitivity; specificity	rifampicin	99.0; 99.0 %	as Base case	as Base case	99.8; 99.8 %
	isoniazid	96.0; 100 % [28]			99.2; 100 %
					(Optimized^a^)
Resistance test for second-line drugs					
Assay		DST on LJ	rapid MRD	rapid MRD	rapid MRD
Specimen		cultured isolate	cultured isolate	directly on sputum	cultured isolate
Accuracy: sensitivity; specificity	fluoroquinolones	100 % (definition)	83.1; 97.7 %	as A	96.6; 99.5 %
	second-line injectable drugs		79.5; 95.8 % [9]		95.9; 99.2 %
					(Optimized^a^)
Treatment regimen individualization					
First-line regimen		Standard regimen	Standard regimen	Standard regimen	Standard regimen
Second-line regimen		Empirical at treatment initiation, individualized after DST result (2+ months)	Empirical at treatment initiation, individualized after culture + MRD result (2+ weeks)	Individualized from treatment initiation	Empirical at treatment initiation, individualized after culture + MRD result (2+ weeks)

Main assumptions: The sensitivity and specificity of molecular tests to detect Rifampicin and INH resistance are the same for all molecular tests (LiPA, MLPA, Xpert MTB/RIF) and are taken as the values of LiPA [29]

*MRD* Molecular Resistance Detection, *DR* drug resistance, *LiPA1* Line Probe Assay for first-line TB drugs, *Xpert* Xpert MTB/RIF assay

2+ =2 or more

^a^Optimized assumes 80 % less false negatives and 80 % less false positives

An overview of the model structure and assumptions is provided below. A full list of model parameters is shown in Tables 2 and 3. Detailed assumptions and outcome definitions are in the Additional file 1.

**Table 2:** Model parameters for cohort proportions, diagnostic test performance and costs

		PE	Range		Source, scenario
Cohort proportions					
	Proportion of PTB patients who are sputum-smear positive	0.64	0.62	0.65	[29] α
	Susceptible to all first line drugs, or resistance to either streptomycin, ethambutol or pyrazinamide, or combinations of those.	0.71	0.75	0.55	[29] α
	IHN mono resistance, which may or may not include resistance to other first line drugs streptomycin, ethambutol, and/or pyrazinamide (poly resistance), but not rifampicin	0.127	0.128	0.122	[29] α
					Footnote (a)
	Rifampicin resistance with- or without INH resistance, without additional resistance to 2nd line drugs. Resistance to ethambutol and/or pyrazinamide may or may not be present.	0.108	0.073	0.212	[29] α
	MDR with additional resistance to ≥1 fluoroquinolone(s) but not to second-line injectable drugs (pré-XDR)	0.008	0.006	0.015	[29] α
	MDR with additional resistance to ≥1 SLID but not fluoroquinolones (pré-XDR)	0.044	0.032	0.087	[29] α
	XDR: MDR with additional resistance to ≥1 fluoroquinolones and ≥1 SLID.	0.008	0.007	0.014	[29] α
Diagnostic accuracy parameters					
	Sensitivity of molecular tests in detecting rifampicin resistance (assumed to be the same as LiPA)	0.99	0.96	1.00	[28] α
	Sensitivity of molecular tests in detecting INH resistance (assumed to be the same as LiPA)	0.96	0.93	1.00	[28] α
	Sensitivity of molecular tests in detecting resistance to fluoroquinolones	0.831	0.787	0.867	[9] δ
	Sensitivity of molecular tests in detecting resistance to SLID, taken as the sensitivity of LiPA sl to detect capreomycin resistance	0.795	0.583	0.914	[9] δ
	Specificity of molecular tests in detecting rifampicin resistance (assumed to be the same as LiPA)	0.99	0.98	1.00	[28] α
	Specificity of molecular tests in detecting INH resistance (assumed to be the same as LiPA)	1.00	0.99	1.00	[28] α
	Specificity of molecular tests in detecting resistance to fluoroquinolones	0.977	0.943	0.991	[9] δ
	Specificity of molecular tests in detecting resistance to SLID, taken as the sensitivity of LiPA sl to detect capreomycin resistance	0.958	0.934	0.973	[9] δ
	Sensitivity and specificity of DST for resistance to 1st and 2nd line drugs	1	-		model assumption β
Repeat testing (Proportion of tests with invalid results requiring repeat testing, for:)					
	Xpert MTB/RIF	0.011	0.0004	0.020	[30] γ
	LiPA	0.027	0.007	0.068	[3] γ
	mycobacterial culture	0.052	0.048	0.057	[31] γ
	high-throughput MRD-assay	0.027			same as LiPA; δ model assumption
	phenotypic DST	0			model assumption; β footnote (b)
Median number of days to result^a^		days	sd		source
	MTBDRplus assay (LiPA)	3.0	1.7		[4]; γ footnote (c)
	LJ culture	34.1	11.3		[4] β
	MGIT culture	8.9	3.9		[4] γ
	LJ DST	67.5	15.0		[4] β
	MGIT DST	21.6	9.3		[4] β
	high-throughput MRD assay in scenarios A and C	6.0	3.0		Model assumption; footnote (d)
	high-throughput MRD assay in scenario B	3.0	1.5		Model assumption; footnote (e)
	Xpert MTB/RIF	0			Model assumption; γ footnote (f)
Median days from lab result until clinical review and treatment initiation					
	for a standard treatment regimen (1st line or empirical 2nd line)	1			[32] α
	for an individualized regimen (assuming additional consultation)	4			Model assumption α
Median days from treatment initiation to sputum culture conversion					
	in patients with susceptible TB or INHmono resistance (days, sd)	34	26		[33–35] α
	in patients with MDR-TB on an appropriate regimen, (days, 95 % CI)	61	59	67	[13] α
	in patients with XDR-TB in high-throughput RMD scenario (days, 95 % CI)	75	60	90	[14] δ
	Increase in duration of préXDR (SLID res) in baseline	0.55			[15] β
	Increase in duration of préXDR (FQ res) in baseline	0.72			[15] β
Time to failure					
	Months to failure on a first-line regimen	5			(15;30) α
	Months to failure on a second-line regimen	4			(15;30) α
	Infectious time in XDR patients who fail	24			Model assumption (duration of treatment) α
Per-test unit cost for diagnostic tests US$ 2013 (min, max)					
	Sputum smear [2]	3.34	2.42	5.08	[17] α
	Xpert PEPFAR pricing	17.29	15.66	18.92	[18] γ
	high-throughput MRD-assay - ratio compared to per-test unit costs of LiPA	2	0.5	4	model assumption δ
	DST 1st line (MGIT)	44.56	40.05	49.07	[18] β
	DST 2nd line (LJ)	25.35	20.68	30.02	[18] β
	Line Probe Assay (LiPA)	21.32	18.45	24.18	[18] γ
	LJ culture	18.48	11.08	33.30	[17] γ
	MGIT culture	18.48	11.08	33.30	[17] γ
Treatment cost parameters US$ 2013 (min, max)					
	First-line treatment course^b^	945	629	1419	[19] α
	Second-line treatment course for MDR	4176	2341	7449	[19] α
	Ratio of pré-XDR regimen cost compared to MDR regimen cost	2			[20] α
	Ratio of XDR regimen cost compared to MDR regimen cost	3			[20] α
	Hospitalization for MDR/XDR, cost per day^b^	67			[1] α

The modeled scenarios are: Base Case; MRD-A. Rapid MRD assay following culture; MRD-B. Improved analytical sensitivity; MRD-C. Improved clinical accuracy. The Greek symbol in the first column indicates to which scenarios the parameter apply: α to all four scenarios; β to the Base Case only; γ to the Base Case, MRD-A and -C but not to MRD–B; δ to the MRD scenarios (A, B, C) but not the Base Case

(a): low end of the range reflects the distribution among new patients and high end the distribution among previously treated patients.(b): if an isolate is obtained on culture, we assume DST will always give a valid result. (c): adjusted (was 4.2 days in the publication in a special study performing assays 2–3 times a run per week on 2–8 samples per run. We adjusted for current practice where LiPAs are run daily (50–60 samples per week). (d) assumes a batch of ± 50 once a week. (e): assumes a patient volume that requires batch testing of ± 50 every 1–2 (working) days. (f): Time counts from TB diagnosis and Rifampicin result comes at the same time as the TB diagnostic result

Abbreviations; *PE* point estimate, *PTB* pulmonary tuberculosis, *MDR* multi-drug resistance, defined as resistance to rifampicin and isoniazid [2], *SLID* second-line injectable drugs, *XDR* extensively drug-resistant, *INH* isoniazide, *LiPA* line probe assay, *LJ* Löwenstein-Jensen, *MGIT* Mycobacterial Growth Inhibitor Tube, *DST* Drug Susceptibility Testing, *sd* standard deviation, *CI* confidence interval

^a^Excluding requirement for repeat testing

^b^First-line treatment applies to catI and catII treatment; hospitalization costs are estimated from studies in the same region [21] and average number of hospital days in 2012 [36]

**Table 3:** Results of the primary analysis for a simulated cohort of 1000 patients diagnosed with TB

	No. of TB patients in cohort	No. with (pre-)XDR	No. (%) of (pre-) XDR diagnosed earlier c.t. base case		Total No. of IPM among cohort; % Change c.t. base case		No. of PNTPM; % Change c.t. base case		% of all IPM	Total cohort costs^a^ (US$ 2013); % change c.t. base case		Diagnostic costs (US$ 2013); % of total costs		Deaths (n; % Change c.t. base case	Need for re-treatment (n)^b^
Base case	1000	59			1,710		66		4 %	$3,557,923	0 %	$44,617	1.2 %	39.1	125
High-throughput MRD-assay:															
A. following culture	1000	59	45	76 %	1,603	−6 %	76	+15 %	5 %	$2,960,243	−17 %	$39,837	1.3 %	40.1 (+2.5 %)	127 (+1.6 %)
B. Improved analytical sensitivity	1000	59	45	76 %	1,617	−5 %	71	+8 %	4 %	$2,821,923	−21 %	$48,816	1.7 %	40.1 (+2.5 %)	129 (+3.9 %)
C. Improved clinical accuracy	1000	59	57	96 %	1,604	−6 %	50	−24 %	3 %	$2,937,299	−17 %	$39,233	1.3 %	40.1 (+2.5 %)	124 (−0.4 %)

*IPM* infectious person-months, *c.t.* compared to, *PNTPM* potential nosocomial transmission person-months; i.e. IPM in (pre-)XDR patients that may cause nosocomial transmission, *MRD* molecular resistance detection

^a^Total costs combine diagnostic, treatment, hospitalization costs

^b^% change is the same for deaths and need for retreatment

### Population

The setting had the epidemiological characteristics of the Republic of Georgia, which is a high-MDR setting with an estimated TB incidence of 116 per 100,000 in 2012, of whom approximately ¾ were patients with pulmonary TB (PTB) [1]. We assumed patients were diagnosed with PTB either by sputum smear microscopy, the Xpert MTB/RIF assay (*Cepheid*, Inc. (Sunnyvale, CA) [Xpert]), or clinically (but presumably detectable by Xpert). TB diagnosis is decentralized, but DST is centralized in one laboratory serving a population of 4.3 million [11]. The cohort combined new and previously treated patients, was divided into sputum smear-positive and smear-negative patients, and into six different drug resistance patterns: pan-susceptible, INH monoresistant, MDR, MDR plus fluoroquinolone-resistant (pre-XDR-F), MDR plus injectable-resistant (pre-XDR-I) and XDR (Table 2). The distribution of drug resistance reflected that of the Georgian TB patient population [7, 11, 12], and took into account a prevalence of 9.2 % (7.9–11 %) MDR-TB among new patients and 31 % (27-35 %) in previously treated patients [11]. HIV-status was not considered.

### Diagnostic scenarios

The four diagnostic scenarios that we compared (Table 1) comprise a base case, and three scenarios included a high-throughput multiplex assay for molecular resistance detection (hereafter ‘high-throughput MRD-assay’) was employed. Treatment was initiated as described in the ‘Treatment’ paragraph.

### Base case

The base case was a simplification of the use of alternative tests as currently done in Georgia. The line probe assay for first-line drug mutations ([LiPA1] GenoType MTBDRplus (*Hain Lifescience* GmbH, Nehren, Germany) and Xpert were used in smear-positive and smear-negative patients respectively to detect resistance against first-line drugs. If rifampicin resistance was found, culture on liquid and solid media was initiated and phenotypic DST was used to confirm drug susceptibility, for first-line drugs in automated mycobacterium liquid growth identification tubes ([MGIT] *BACTEC MGIT 960*, *Becton Dickinson* [*BD*] Biosciences, Sparks, MD) and for second-line drugs on *Löwenstein–Jensen* medium (LJ).

A. Rapid test following culture
  This scenario employed a high-throughput MRD-assay that can be used on cultured isolates but not directly on clinical specimens (e.g. sputum). The clinical sensitivity and specificity [10] of the assay for each drug were as shown in Table 1. MTB isolates obtained from MGIT culture were tested with the high-throughput MRD-assay to detect resistance mutations for second-line drugs, thus replacing phenotypic DST for second-line drugs in the base case. Since obtaining isolates takes time [4], first-line drug resistance testing was performed with the same rapid standard of care tests (LiPA1 or Xpert) as applied in the base case.
B. Improved analytical sensitivity
  This scenario employed the assay of scenario A, but hypothesized to have optimized analytical sensitivity so that it could be applied directly on clinical specimens (sputum) of both smear-positive and smear-negative Xpert-positive patients, and thereby replaced all other tests for DR detection of first- and second-line drugs. The accuracy of the high-throughput MRD-assay in identifying clinical resistance against first-line and second-line drugs was the same as in scenario A.
C. Improved clinical accuracy
  This scenario employed the assay of scenario A, but hypothesized to have optimized accuracy in identifying clinical resistance, e.g. by adding additional molecular markers. Since 100 % sensitivity and specificity may be unattainable we simulated that the sensitivity and specificity would improve by 80 % towards the target of 100 %, implying that for each drug in the model the proportions false-negatives and false-positives reduced by 80 % (see Additional file 1). Analytical sensitivity was the same as in scenario B, so cultured isolates were required and LiPA1 and Xpert were used for first-line resistance testing.

We assumed that the sensitivity and specificity of molecular tests for detecting rifampicin and isoniazid resistance were the same for all molecular tests LiPA1, Xpert, and high-throughput MRD (except in the optimized clinical accuracy scenario B) and were taken as the published values of LiPA1 [5]. The sensitivity determined the number of drug-resistant cases that are correctly identified by the test, and specificity the number of patients treated for resistance due to a false positive test result. This implied that all molecular methods in the base case, and high-throughput MRD scenarios A and C detect and miss the same cases compared to phenotypic DST. The accuracy of the high-throughput MRD-assay in detecting mutations conferring resistance to second-line drugs equaled that of second-line LiPA [10], as these values are currently achievable with a molecular test. Phenotypic DST was taken as the reference standard, implying a sensitivity and specificity equal to 100 % for all resistance patterns. Additional details on diagnostic assumptions are provided as Additional file 1.

### Treatment assumptions

Treatment initiation was according to test results, regardless of a prior history of TB treatment: A standard 6 month first-line regimen if results showed susceptible-TB, 9 months if in the INH-mono-resistance category [6]. In the base case and in high-throughput MRD scenarios A and C an empirical standardized second-line regimen was initiated if rifampicin resistant [6]. The empirical second-line regimen was adjusted to an individualized regimen, if needed, once the full resistance profile was known. In scenario B (optimized analytical sensitivity) second-line treatment was individualized from the onset.

The probabilities of treatment outcomes (cure/completion, failure, default or death) depended on the treatment regimen and its adequacy for the drug susceptibility pattern (Additional file 1). In case of treatment failure, patients were retested according to the scenario and switched to an alternative regimen if an underlying resistance pattern had been misdiagnosed earlier on, as further described in the Additional file 1.

We modeled resistance to the most important drugs in second-line treatment each as one group implying that if resistance to one drug in the category is present, none of the drugs in the category were assumed to be effective.

### Costs

Costs were divided into diagnostic costs for TB bacteriological tests, and treatment costs which included hospitalization, and drugs and additional costs like treatment monitoring. All costs were taken from the literature (Table 2) and converted to US$ 2013 [22]. In the primary analysis we assumed the per-test unit-cost of the high-throughput MRD-assay to be twice that of LiPA1 and explored a wider range in a sensitivity analysis. Hospitalization costs assumed that MDR/(pre-)XDR patients were hospitalized during their IPMs. Patients on first-line regimens did not accrue hospitalization costs, since these costs were small compared to MDR/(pre-)XDR and the same in all scenarios [1].

### Analysis

We reported all model outcomes assuming a cohort simulation of 1000 patients diagnosed with TB. In the primary analysis the point estimates (PE) of all parameter values (Table 2) were used. We conducted deterministic sensitivity analyses to explore the effect of uncertainty in the values of key parameters and the effect of assumptions on the primary outcomes (total costs and PNTPM) and on diagnostic costs per (pre-)XDR patient identified, as outlined in the Additional file 1.

### Ethical approval

Ethical approval was not sought as only secondary data were used.

## Results

### Primary analysis

#### Infectious period of time

Following the distribution of drug-resistance patterns, our simulated cohort of 1000 patients diagnosed with TB had 59 patients with (pre-)XDR, detectable by phenotypic DST. In MRD scenarios A and B, 45 (76 %) were correctly identified at an earlier point in time compared to the base case, and 57 (96 %) in scenario C (Table 3). The remaining patients were identified after treatment failure. The number of IPM in (pre-)XDR patients that may lead to nosocomial transmission was 66 in the base case, and reduced by 24 % in scenario C, but increases by 15 % and 7 % in scenarios A and B, respectively, due to FQ and/or SLID resistance patterns having been missed in some of the patients. The total number of IPM in in the cohort was 1710 in the base case, and reduced by 5–6 % in all three MRD scenarios.

#### Cost

The total costs to test for drug resistance and treating all 1000 TB patients in the base case were $3,557,923 of which costs for diagnostic tests comprised 1.3 %. Total costs in the MRD scenarios reduced by between 17–21 %, almost entirely due to a reduction in cost for treatment and hospitalization. Diagnostic costs were highest in scenario B where the costs for the molecular assay applied to all patients, but remained a small fraction of the combined diagnostic, treatment and hospitalization costs. The projected number of deaths and retreatment cases in scenario C remained the same as in the base case, and increased by 2.5 % in the MRD scenarios due to patients starting on an inappropriate treatment regimen.

### Deterministic sensitivity analysis

**Fig. 2:**
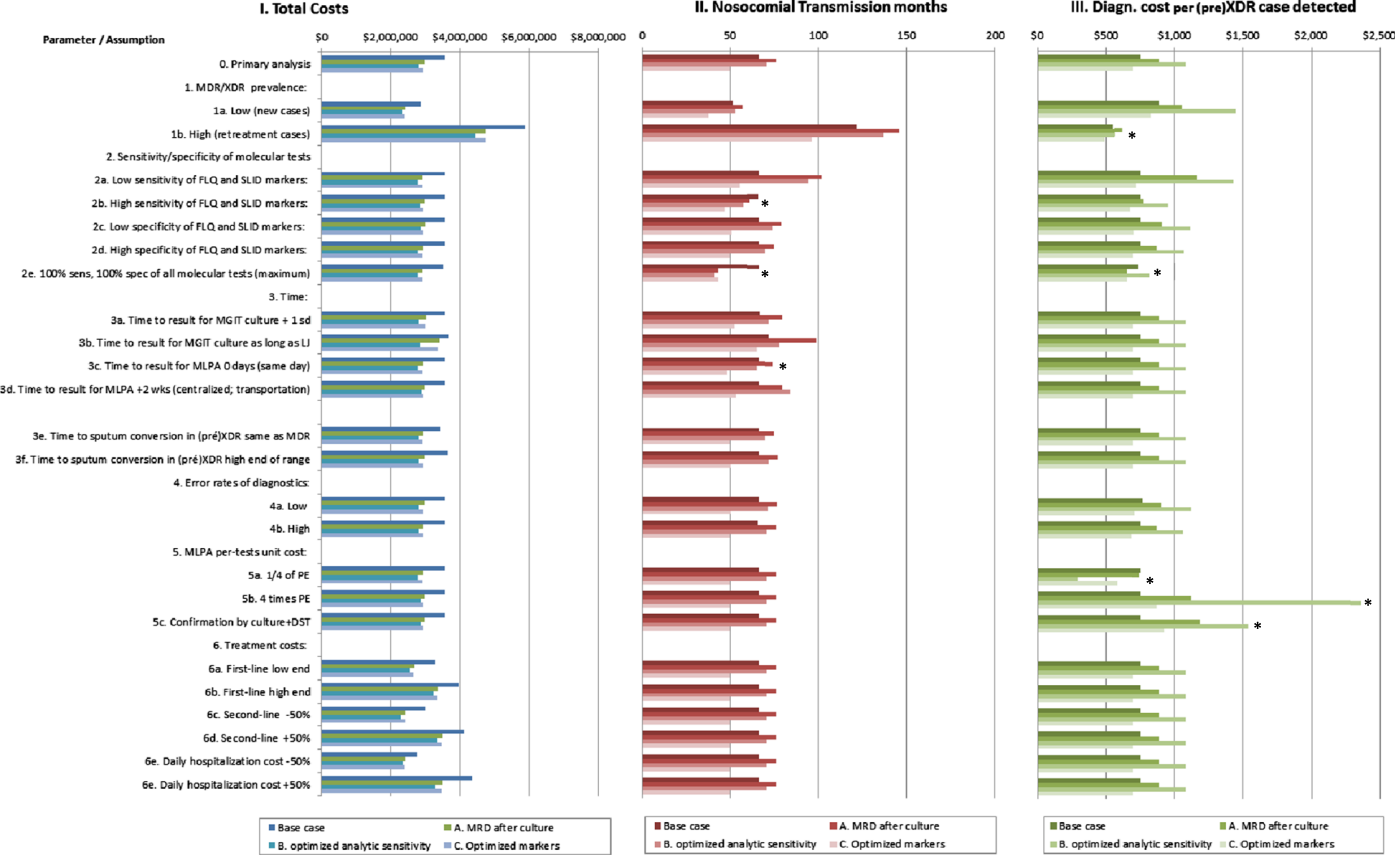
One-way sensitivity analysis showing the magnitude of the effect of each listed parameter or assumption on Total costs, Nosocomial transmission months and Diagnostic cost per (pre-)XDR case detected. Legend: MRD = molecular resistance detection, FLQ = fluoroquinolones, SLID = injectable aminoglycosides, MDR = multi-drug resistance, defined as resistance to rifampicin and isoniazid, XDR = extensively drug-resistant. * indicates a change in ranking

The largest variation (Fig. 2; Additional file 2: Table S1) in total costs was caused by variation in the prevalence of MDR and (pre-)XDR in the cohort (Fig. 2 - Panel I), followed by variation in treatment and hospitalization costs. The ranking of the scenarios in terms of total costs did not change in any sensitivity analysis. Diagnostic costs per (pre-)XDR patient identified (Panel III) were most sensitive to assumptions about the per-test costs of the high-throughput MRD-assay, especially for scenario B. If this cost increased or decreased by 200 %, the diagnostic costs per (pre-)XDR patient in scenario B changed accordingly and became lowest or highest of all scenarios. The effect on total costs remained however between −1 to +2 %, negligible compared to effects of variation in treatment and hospitalization costs. If treatment and hospitalization costs increased or decreased by 50 %, total costs in all scenarios increased or decreased by 16–22 %.

**Fig. 3:**
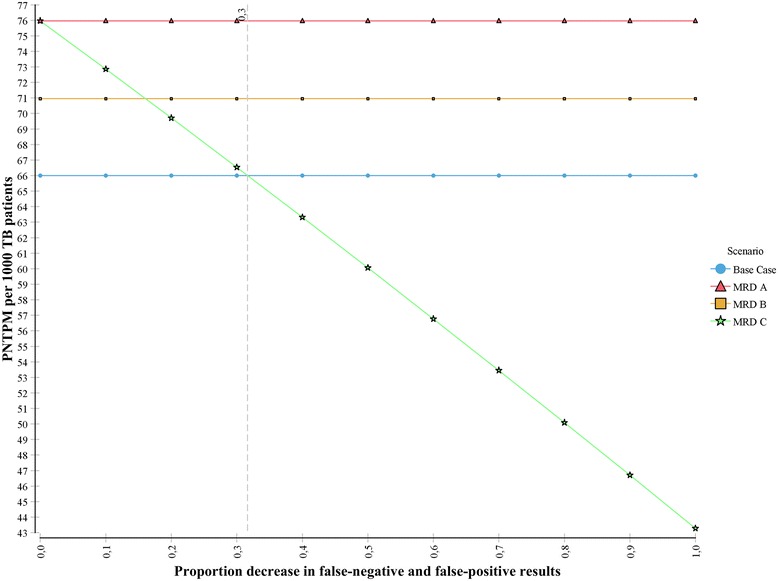
The effect of variation in improvement in the clinical sensitivity and specificity of the assay molecular markers in scenario C (improved markers) on potential nosocomial transmission person months. Legend: The horizontal axis reflects the proportional decrease in false-negative (FN) and false-positive (FP) results (reflecting improvement in clinical accuracy) for second-line resistance in scenario MRD C. The vertical axis represents the number of potential nosocomial transmission person months (PNTPM) per 1000 TB patients in the simulated cohort. Scenario MRD A. represents the MRD assay following culture; MRD B. Improved analytical sensitivity; In scenarios MRD A. and B. the sensitivity and specificity are as reported in the primary analysis. In scenario MRD C. (improved clinical accuracy) the default proportion reduction in FN and FP results was 0.8 in the primary analysis. The vertical dotted line represents the minimum decrease in the proportion FN and FP that is required to ensure that PNTPM in scenario MRD C are at least equal to the Base case. The PNTPM in scenarios MDR A and B exceed that of the Base case, reflecting greater potential for nosocomial transmission

The number of PNTPM (Fig. 2 - Panel II) was, as expected, also most sensitive to variation in the prevalence of MDR and (pre-)XDR in the cohort, followed by the sensitivity of the assays for detecting FQ and SLID resistance. In scenario C (optimized markers) the number of PNTPM would fall below the base case level if the proportion of false-negatives and false-positives reduced by at least 35 % (Fig. 3). If assay sensitivity was set at 100 % (equal to phenotypic DST), rapid DR testing reduced nosocomial transmission time by more than half.

If we assumed that the time to obtain a cultured isolate for high-throughput analysis increased to the days required for LJ culture, PNTPM increased by 30 % compared to the use of MGIT in scenarios A and C, which first required culture. The effect of variation in assumptions about the time to sputum conversion in (pre-)XDR, about error rates of diagnostic procedures (contaminated cultures etc.), and of increased turnaround time by 2 weeks (e.g. due to transportation of specimens) was small and changed the number of PNTPM on the order of 2–8 % compared to the primary analysis. If we assumed that confirmatory phenotypic DST (first- and second-line) would be done in addition to rapid MDR in scenarios A, B and C to avoid increase in retreatment and death, diagnostic cost increased by 33–42 % and comprised 1.8–2.4 % of total cohort costs which increased by 0.4–2 %.

## Discussion

We conclude from our model that introducing a high-throughput MRD-assay as the primary diagnostic test for faster detection of resistance-conferring mutations for second-line anti-tuberculosis drugs could potentially reduce the combined costs for diagnosis, treatment and hospitalization of TB patients by 17–21 %. Due to the low clinical sensitivity, the use of molecular assays for second-line drug resistance may however have perverse consequences in terms of nosocomial transmission in settings where MDR and (pre-)XDR patients are hospitalized until sputum culture conversion has been confirmed. A longer hospitalization period of missed cases of (pre-)XDR patients may increase opportunities for nosocomial transmission to patients infected by MDR-TB that is susceptible to second-line drugs.

In terms of improving the performance of high-throughput MRD-assays, our results suggest that greater impact on reducing infectious time in general and potential for nosocomial transmission is expected from increasing clinical sensitivity and specificity (optimized markers) than from optimizing analytic sensitivity (allowing direct analysis of sputum) without improvement of the markers. This still applies if the turnaround time of an improved assay requires MGIT culture and 1–2 weeks to send specimens and return results to peripheral facilities. We found that costs for diagnostic tests remain a small proportion of total costs for diagnosis and treatment combined, even if the average per-test costs of MRD doubled compared to our primary assumption.

The rapid second-line assay that we modelled combines high-throughput features of the MLPA [8] with published accuracy of second-line LiPAs [10]. A pilot has demonstrated operational feasibility of the MLPA technology in a centralized reference laboratory in a high MDR-TB setting to analyze batches of cultured isolates (Sengstake et al. manuscript in preparation). The clinical accuracy of the prototype assay in detecting molecular resistance to first and second-line drugs is under evaluation, and is influenced by the composition of genetic markers targeted. For further development of the high-throughput bead-based MLPA technology [8] that served as the example for this study, our results suggest that further investments should first be in improving markers to reach a sensitivity beyond the values that we used in this modeling study. A possible increase in per-test unit costs of an improved assay would be outweighed by reductions in treatment costs. Investment towards an improved high-throughput assay for centralized use may be preferred above investment in improved rapid assays that allow decentralized detection of second-line mutations directly from sputum, as such a test would require excellent analytic sensitivity in smear-positive as well as smear-negative sputum samples. As long as genetic targets are sufficiently tailored to the local epidemic drug resistant clusters, the MLPA technology or any other similar MRD test would be advantageous [23]. Increasing clinical sensitivity should not compromise specificity. In low MDR-TB settings excellent specificity would also be needed to avoid false-positive diagnosis of M(X)DR-TB.

Our study has a number of limitations. We made simplifying assumptions, such as that a multiplex molecular assay would replace phenotypic DST for diagnosis of second-line drug resistance. A single test would reduce the costs and complexity of post-TB-diagnosis analysis, including the cost related to the development of phenotypic DST laboratory capacity (not included in our calculations), and may thereby allow the further scale-up of second-line treatment in resource-poor settings needed to treat a larger proportion of M/XDR-TB patients. We acknowledge that the reality of diagnosis and treatment of MDR-TB is complex [24]. For clinical decision making, genotypic assays with low sensitivity may be used alongside phenotypic DST in practice [9]. Our results show that confirmation of second-line resistance with phenotypic DST should remain mainstay unless the sensitivity of molecular markers improves, to avoid unfavorable effects on infection control and patient outcomes. In any case regular validation with phenotypic DST and clinical response remains required, since adding more markers will only improve sensitivity, if new strains (carrying new drug resistance conferring mutations) are introduced in the setting.

The assumption that if resistance to one drug in the category is present, none of the drugs in the category will be effective may not always be correct. Newer generation fluoroquinolones such as moxifloxacin may be effective when there is resistance to older generation fluoroquinolones such as ofloxacins [25]. The meta-analysis from which we sourced the MTBDR*sl* test characteristics [10] included both studies that had DST for ofloxacin and studies that had DST for moxifloxacin as the reference standard, so our parameter values were a composite for both drugs. Specificity for moxifloxacin resistance was somewhat lower, and sensitivity somewhat higher, than for ofloxacin, so we may have overestimated the effects on infectious person-time, and underestimated the effects on unnecessary treatment changes if newer generation fluoroquinolones would be used. Similarly, sensitivity of molecular diagnosis is lower for kanamycin than for amikacin, and similar effects may have occurred for the category of SLID. Nonetheless, such variations would not affect the conclusion that a beneficial effect of a MRD test requires high clinical sensitivity.

Our decision modeling approach did not take transmission other than nosocomial into account and we did not make inferences about potentially reduced transmission due to the reduction of infectious time. Transmission depends on many additional factors, including infectious time prior to TB diagnosis and in persons who default treatment, which are amenable to other types of interventions.

Additional benefits of second-line MRD tests were not included in the analysis, a simplification that avoids overestimation of the impact of rapid second-line resistance testing. Additional benefits include a targeted regimen with fewer drugs compared to the empirical regimen. This may also reduce side effects and toxicities and chances of treatment default [26], and prevent resistance amplification, i.e. acquired resistance against additional classes of second-line drugs [7]. Prevention of amplification may further reduce costs by preventing treatment failure. Another additional benefit may be less pre-treatment loss to follow-up compared to phenotypic DST as a result of much shortened time-to-result. As this study pertains to patients who are hospitalized after TB diagnosis, effects on pre-treatment loss to follow-up were not considered.

Our approach is novel in that we modeled IPM and the potential of nosocomial transmission as outcomes since those factors are direct concerns in settings with high prevalence of MDR-TB and (pre-)XDR-TB. The purpose of this study is to show potential trends and should not be interpreted as a cost-effectiveness study for one particular setting and technology. A recent modeling study suggested that to be potentially cost-effective in terms of preventing mortality and disability, the aggregate sensitivity and specificity of multiplex assays for pre-XDR/XDR should at least be 88 and 96 %, respectively [27]. Empirical data to support model assumptions about mortality and disability arising from inadequate treatment of pre-XDR/XDR-TB are however scarce [27]. Although this study and ours each compass its own uncertainty in assumptions, both approaches point in the same direction.

## Conclusions

A high-throughput MRD-assay for early detection of resistance to second-line drugs as a replacement of phenotypic DST could potentially reduce the combined costs for diagnosis, treatment and hospitalization of TB patients and may seem attractive for infection control purposes. Low sensitivity may however compromise infection control in settings where MDR and (pre-)XDR patients are hospitalized, and affect patient outcomes unfavorably. Further investments to improve the overall sensitivity are needed with a priority for improvements in clinical above analytical sensitivity.

## Additional files

Additional file 1:
**Expanded Methods and model parameters of treatment outcomes.** (DOCX 70 kb) Additional file 2: Table S1. Results of deterministic sensitivity analyses (XLSX 20 kb)

## Abbreviations

DST: Drug susceptibility testing (phenotypic)
FQ: Fluoroquinolones
HIV: Human immunodeficiency virus
IPM: Infectious person-months
LiPA: Line-probe assay
LiPA1: Line-probe assay to detect first-line drug mutations
LJ: Löwenstein–Jensen medium
MDR: Multidrug-resistant
MGIT: Mycobacterium liquid growth identification tube
MLPA: Multiplex Ligation-dependent Probe Amplification assay
MRD: Molecular resistance detection
MTB: Mycobacterium tuberculosis
PNTPM: Potential nosocomial transmission person months
pre-XDR-F: MDR plus fluoroquinolone-resistant
pre-XDR-I: MDR plus injectable-resistant
PTB: Pulmonary tuberculosis
SLID: Second-line injectable drugs
TB: Tuberculosis
XDR-TB: Extensively drug resistant tuberculosis
Xpert: Xpert MTB/RIF assay
