# Supplementary material for: The potential of a multiplex high-throughput molecular assay for early detection of first and second line tuberculosis drug resistance mutations to improve infection control and reduce costs: a decision analytical modeling study
Source: BMC Infect Dis. 2015 Oct 26;15:473. doi: 10.1186/s12879-015-1205-4 (PMC4624169; doi:10.1186/s12879-015-1205-4)
Supplement: Additional file 1: — Expanded Methods and model parameters of treatment outcomes. (DOCX 70 kb) [file 12879_2015_1205_MOESM1_ESM.docx]

**SUPPLEMENTAL DOCUMENT – EXPANDED METHODS**

**LIST OF ABBREVIATIONS AND DEFINITIONS**

| DST | drug susceptibility testing (phenotypic) |
| --- | --- |
| FQ | fluoroquinolones |
| HIV | human immunodeficiency virus |
| IPM | infectious person-months |
| LiPA | Line-probe assay |
| LiPA1 | Line-probe assay to detect first-line drug mutations |
| LJ | Löwenstein*–*Jensen medium |
| MDR | multidrug-resistant |
| MGIT | mycobacterium liquid growth identification tube |
| MLPA | Multiplex Ligation-dependent Probe Amplification assay |
| MRD | Molecular resistance detection |
| MTB | *Mycobacterium tuberculosis* |
| PNTPM | Potential nosocomial transmission person months |
| pre-XDR-F | MDR plus fluoroquinolone-resistant |
| pre-XDR-I | MDR plus injectable-resistant |
| PTB | pulmonary tuberculosis |
| SLID | second-line injectable drugs |
| TB | tuberculosis |
| XDR-TB | extensively drug resistant tuberculosis |
| Xpert | Xpert MTB/RIF assay |
|  |  |
| **Definitions** |  |
| Analytical sensitivity | The smallest amount of substance in a sample that can accurately be  measured by an assay (1) |
| Clinical (or Diagnostic) sensitivity | Percentage of persons who have a given disorder who are identified by the assay as positive for the disorder (1) |

**METHODS EXPANDED**

***Population***

*We made the following simplifying assumptions with respect to the population:*

- The distribution of resistance patterns was the same in smear-positive and smear-negative patients.
- All TB cases are HIV-negative. Since HIV prevalence is low in this setting (2) HIV-status was not considered in the model.
- Resistance to ethambutol and/or pyrazinamide may be present but did not affect the outcomes.

***Diagnostic scenarios***

*Additional information with respect to scenario C. Improved clinical accuracy*

This scenario employed the assay of scenario A, but hypothesized to have optimized accuracy in identifying clinical resistance, e.g. by adding additional molecular markers. Since 100% sensitivity and specificity may be unattainable we simulated that the sensitivity and specificity would improve by 80% towards the target of 100%, implying that for each drug in the model the proportions false-negatives and false-positives reduced by 80% (see Supplement). For example the sensitivity to detect FQ resistance was 83.1% in scenario B. If the proportion false-negatives (100%-sensitivity) of 16.9% would reduce by 80%, to 3.4%, the sensitivity of the assay would improve to 96.6%. Similarly, 80% reduction of false-positives (100%-specificity) would increase specificity from 97.7% to 99.5%. Analytical sensitivity was the same as in scenario B, so cultured isolates were required and LiPA1 and Xpert were used for first-line resistance testing. Analytical sensitivity was the same as in scenario B, so cultured isolates were required and LiPA1 and Xpert were used for first-line resistance testing.

*We made the following assumptions with respect to the diagnostic scenarios*

- The sensitivity and specificity of molecular tests for detecting rifampicin and isoniazid resistance were the same for all molecular tests LiPA1, Xpert, and high-throughput MRD (except in the optimized clinical accuracy scenario B) and were taken as the published values of LiPA1 (3). This implied that all molecular methods in the Base case, and high-throughput MRD scenarios A and C detect and miss the same cases compared to phenotypic DST.
- The accuracy of the molecular assay in detecting mutations conferring resistance to second-line drugs equaled that of second-line LiPA (4), as these values were currently achievable with a molecular test.
- Phenotypic DST was taken as the reference standard, implying we assumed it had a sensitivity and specificity equal to 1 for all resistance patterns.
- For the molecular assay we considered second-line drugs in one class together. For SLID we took the sensitivity and specificity of capreomycin (4) and assumed that this reflected the sensitivity and specificity of kanamycin, amikacin and the polypeptide capreomycin combined.
- Likewise a FQ resistant result reflected clinical resistance to all fluoroquinolones, including the later generations.
- All patients in the cohort had disease caused by *Mycobacterium tuberculosis* (MTB) that could be detected by Xpert and yielded an isolate on culture.
- All tests produce erroneous results at pre-specified rates (5-7), requiring repeat testing after which a valid result was obtained. The detection of DR for each drug-class was independent, i.e. probabilities were multiplied.
- Poly-drug resistance to other first-line drugs but not rifampicin is considered to be the same as for isoniazid mono resistance.
- Although a high-throughput MRD-assay as the primary test would allow for unexpected combinations of drug resistance patterns, the model did not allow for rifampicin sensitive patients to have (false) positive FQ and SLID results. Given the high specificity of molecular assays (3;5;8) the probability of 2 consecutive false-positive tests was considered highly unlikely.

***Additional Treatment assumptions***

*Treatment initiation*

In the base case and in high-throughput MRD scenarios A and C treatment was initiated according to the LiPA1 or Xpert result, regardless of a prior history of TB treatment. Patients categorized as ‘susceptible’ received a standard first-line regimen as used for new patients (‘*2RHEZ 4RH’*), and patients in the category INH-mono-resistance a standard category II regimen for 9 months (9). Patients were initiated on an empirical standardized second-line regimen if rifampicin resistant (9). Once the full resistance profile was known the empirical second-line regimen was adjusted to an individualized regimen if needed. In scenario B (optimized clinical sensitivity) first-line treatment was as described above, and second-line treatment was individualized from the onset. We assumed that a patient received an effective treatment regimen based on knowledge of the drug resistance pattern, without specifying what that regimen was.

*Treatment outcomes*

Treatment outcomes were cure/completion, failure, default or death. Probabilities of treatment outcomes depended on the treatment regimen and its adequacy for the drug susceptibility pattern (Table at the end of this supplement).

- We assumed that the probability of default is based on the regimen, considering that regimen-related side effects and anticipated duration are determinants of default.
- The probability of death and failure in patients with susceptible TB on a second-line regimen were based on the regimen, since a second-line regimen is less effective than first-line (10-12).
- The treatment outcome probabilities of patients with MDR/(pre-)XDR TB were assumed to be the same as in XDR patients if treated with non-effective drugs.
- The occurrence of relapse in cured patients was not considered in the model.
- The probability of failure and death of patients with susceptible TB and of patients in the category INHmono/PDR (10) on a first-line treatment regimens were weighed to included 7% retreatment cases and 93% new TB patients (2).
- We assumed that the first-line category I and first-line category II treatment regimens for patients susceptible TB and first-line category II treatment for patients in the category INHmono/PDR are equally effective, resulting in the same probability of failure and of death.

*Treatment failure*

In case of treatment failure, patients on a first-line regimen in the high-throughput MRD scenarios A and C scenarios received a LiPA1 for first-line DR testing, and second-line DST/DR-testing according to the pathway. Underlying MDR/(pre-)XDR patterns in failure patients who were misclassified as not having MDR/(pre-)XDR at onset of treatment were recognized at this stage and these patients were switched to an appropriate regimen. In high-throughput MRD scenario B all failure patients were tested with the high-throughput MRD-assay, presumed to yield results. Treatment failure in patients treated for rifampicin susceptible TB was defined as a TB patient whose sputum smear or culture was positive at month 5 or later (13;14). After DST/DR-testing, category II treatment was started for 9 months. Treatment for MDR-TB or (pre-)XDR-TB was assumed to be monitored according to the guidelines (9) and failure was defined as lack of conversion of the month 4 culture (9;13;14), when patients were switched to a presumably appropriate regimen for another 24 months.

***Outcomes***

*Infectious person-months (IPM) - overall*

The overall IPM, reflecting the period that patients are in care after TB diagnosis until sputum cultures converted to negative, was composed of time for a test to generate results in the laboratory (15;16), time from laboratory result until clinical review and treatment initiation (16) which was longer for individualized second-line treatment since it requires specialist consultation, and median time from treatment initiation to sputum culture conversion for a respective drug resistance pattern (17-19) (Table 2). Specifically:

- Patients who failed on treatment were assumed to remain infectious until failure was established (20) as described above, and an appropriate regimen for the drug-susceptibility pattern resulted in sputum conversion following the same time periods as described in Table 2.
- XDR patients remained infectious as long as their drug resistance pattern is unknown, so more rapid second-line DST results shortened time to sputum conversion accordingly.
- In pre-XDR patients on empirical treatment, time to culture conversion was prolonged compared to ‘MDR-only’ by 72% if FQ resistant and by 55% if SLID resistant (19).
- If patients started individualized treatment immediately (high-throughput MRD scenario B) this was assumed to be as effective as appropriate treatment in ‘MDR-only’ patients in achieving sputum conversion.

*Nosocomial transmission months (primary outcome)*

IPM during which nosocomial transmission of (pre-)XDR could potentially take place applied only to patients with (pre-)XDR-TB. It was taken as the period from TB diagnosis until the moment that the correct drug-resistance pattern was identified, when effective infection control measures could presumably be taken. For (pre-)XDR patients who failed treatment based on the month-4 culture, we assumed that effective infection control measures were undertaken once failure was established.

*Meaningful change of treatment regimen*

As an additional secondary effect outcome we calculated the proportion of pre-XDR and XDR cases correctly identified out of the total number of pre-XDR and XDR cases in the cohort. In the high-throughput MRD scenarios those would benefit from a meaningful change of treatment regimen at an earlier point in time than would have occurred with phenotypic DST. A treatment regimen that required a modification other than a simplification after DST results were available was considered inadequate.

*Number of patients requiring future retreatment, and death*

We assume that failure and default patients required retreatment, but do not die within the modeling timeframe.

*Analysis*

We reported all model outcomes for the point estimates (PE), and conducted deterministic sensitivity analyses to explore how the primary outcomes (total costs and nosocomial transmission months) as well as diagnostic costs per (pre-)XDR patient identified were affected by uncertainty in assumptions and values of key parameters. To explore the effect of variation in the distribution of drug resistance patterns, the patterns in new and previously treated patients were taken as the lower and upper limits of the uncertainty range, respectively. We explored the effect of uncertainty in the performance of diagnostic tests by varying sensitivity and specificity of the FQ and SLID resistance markers, as well as in the error rates of diagnostic tests and procedures. We furthermore examined the effect of time to result for MGIT culture, assumptions about time to sputum conversion, and the time effect of centralization (longer time to lab results), variation of costs parameters for diagnostics, treatment, hospitalization, as well as the duration of treatment in patients who died or defaulted.

Reference List

(1) Saah AJ, Hoover DR. "Sensitivity" and "specificity" reconsidered: the meaning of these terms in analytical and diagnostic settings. Ann Intern Med 1997 Jan 1;126(1):91-4.

(2) World Health Organization. Global Tuberculosis Report 2013. Geneva: World Health Organization (WHO/HTM/TB/2013.11); 2013.

(3) Bwanga F, Hoffner S, Haile M, Joloba ML. Direct susceptibility testing for multi drug resistant tuberculosis: a meta-analysis. BMC Infect Dis 2009;9:67.

(4) Feng Y, Liu S, Wang Q, Wang L, Tang S, Wang J, et al. Rapid diagnosis of drug resistance to fluoroquinolones, amikacin, capreomycin, kanamycin and ethambutol using genotype MTBDRsl assay: a meta-analysis. PLoS One 2013;8(2):e55292.

(5) Tukvadze N, Bablishvili N, Apsindzelashvili R, Blumberg HM, Kempker RR. Performance of the MTBDRsl assay in Georgia. Int J Tuberc Lung Dis 2014 Feb;18(2):233-9.

(6) Steingart KR, Sohn H, Schiller I, Kloda LA, Boehme CC, Pai M, et al. Xpert(R) MTB/RIF assay for pulmonary tuberculosis and rifampicin resistance in adults. Cochrane Database Syst Rev 2013;1:CD009593.

(7) den Hertog AL, Daher S, Straetemans M, Scholing M, Anthony RM. No added value of performing Ziehl-Neelsen on auramine-positive samples for tuberculosis diagnostics. Int J Tuberc Lung Dis 2013 Aug;17(8):1094-9.

(8) Steingart KR, Sohn H, Schiller I, Kloda LA, Boehme CC, Pai M, et al. Xpert(R) MTB/RIF assay for pulmonary tuberculosis and rifampicin resistance in adults (updated). Cochrane Database Syst Rev 2014;1:CD009593.

(9) World Health Organization. Guidelines for the programmatic management of drug-resistant tuberculosis, 2011 update. 2011. World Health Organization.

(10) Espinal MA, Kim SJ, Suarez PG, Kam KM, Khomenko AG, Migliori GB, et al. Standard short-course chemotherapy for drug-resistant tuberculosis: treatment outcomes in 6 countries. JAMA 2000 May 17;283(19):2537-45.

(11) Falzon D, Gandhi N, Migliori GB, Sotgiu G, Cox HS, Holtz TH, et al. Resistance to fluoroquinolones and second-line injectable drugs: impact on multidrug-resistant TB outcomes. Eur Respir J 2013 Jul;42(1):156-68.

(12) Ahuja SD, Ashkin D, Avendano M, Banerjee R, Bauer M, Bayona JN, et al. Multidrug resistant pulmonary tuberculosis treatment regimens and patient outcomes: an individual patient data meta-analysis of 9,153 patients. PLoS Med 2012;9(8):e1001300.

(13) World Health Organization. Revised definitions and reporting framework for tuberculosis. 2013.

(14) World Health Organization. Guidelines for the programmatic management of drug-resistant tuberculosis, Emergency update, 2008. 2008. Geneva, World Health Organization.

(15) Tukvadze N, Kempker RR, Kalandadze I, Kurbatova E, Leonard MK, Apsindzelashvili R, et al. Use of a molecular diagnostic test in AFB smear positive tuberculosis suspects greatly reduces time to detection of multidrug resistant tuberculosis. PLoS One 2012;7(2):e31563.

(16) Rabin AS, Kuchukhidze G, Sanikidze E, Kempker RR, Blumberg HM. Prescribed and self-medication use increase delays in diagnosis of tuberculosis in the country of Georgia. Int J Tuberc Lung Dis 2013 Feb;17(2):214-20.

(17) Conde MB, Efron A, Loredo C, De Souza GR, Graca NP, Cezar MC, et al. Moxifloxacin versus ethambutol in the initial treatment of tuberculosis: a double-blind, randomised, controlled phase II trial. Lancet 2009 Apr 4;373(9670):1183-9.

(18) Gonzalez-Montaner LJ, Natal S, Yongchaiyud P, Olliaro P. Rifabutin for the treatment of newly-diagnosed pulmonary tuberculosis: a multinational, randomized, comparative study versus Rifampicin. Rifabutin Study Group. Tuber Lung Dis 1994 Oct;75(5):341-7.

(19) Holtz TH, Sternberg M, Kammerer S, Laserson KF, Riekstina V, Zarovska E, et al. Time to sputum culture conversion in multidrug-resistant tuberculosis: predictors and relationship to treatment outcome. Ann Intern Med 2006 May 2;144(9):650-9.

(20) Pooran A, Pieterson E, Davids M, Theron G, Dheda K. What is the cost of diagnosis and management of drug resistant tuberculosis in South Africa? PLoS One 2013;8(1):e54587.

| Table. Model parameters for treatment outcomes | | | | | | | | | | | | |
| --- | --- | --- | --- | --- | --- | --- | --- | --- | --- | --- | --- | --- |
|  | **Outcome:** | **Default*** | | |  | **Failure** | | | **Death** | | | **Source** |
| **Treatment category** | **Drug resistance pattern** | **PE** | **low** | **high** | **Source** | **PE** | **low** | **high** | **PE** | **low** | **high** |  |
| First-line category I | Any | 0.059 | 0.052 | 0.066 | (10) |  |  |  |  |  |  |  |
|  | susceptible |  |  |  |  | 0.028 | 0.020 | 0.036 | 0.024 | 0.021 | 0.035 | (10) † |
|  | INHmono/PDR |  |  |  |  | 0.038 | 0.023 | 0.053 | 0.028 | 0.015 | 0.040 | (10) † |
| First-line category II | Any | 0.13 | 0.083 | 0.19 | (10) |  |  |  |  |  |  |  |
|  | susceptible or IHNmono/PDR | | |  |  | 0.028 | 0.020 | 0.036 | 0.028 | 0.021 | 0.035 | (10) † |
| First-line category I or II | MDR/XDR |  |  |  |  | 0.26 | 0.00 | 0.99 | 0.14 | 0.03 | 0.26 | (12) |
| Second-line including FQ and SLID | Any | 0.19 | 0.13 | 0.26 | (11)** |  |  |  |  |  |  |  |
|  | "MDR only", susceptible or IHNmono/PDR | | | | | 0.04 | 0.02 | 0.06 | 0.09 | 0.05 | 0.12 | (11)** |
|  | (pre-)XDR |  |  |  |  |  |  |  |  |  |  |  |
| Second-line including FQ but not SLID | Any | 0.17 | 0.08 | 0.26 | (11)** |  |  |  |  |  |  |  |
|  |  |  |  |  |  | 0.13 | 0.10 | 0.16 | 0.09 | 0.03 | 0.15 | (11)** |
| Second-line including SLID but not FQ | Any | 0.13 | 0.01 | 0.26 | (11)** |  |  |  |  |  |  |  |
|  |  |  |  |  |  | 0.20 | 0.16 | 0.24 | 0.12 | 0.03 | 0.21 | (11)** |
| Second-line excluding SLID and FQ | Any | 0.18 | 0.09 | 0.27 | (11)** | 0.25 | 0.17 | 0.31 | 0.17 | 0.09 | 0.26 | (11)** |
| Any inadequate second-line regimen | MDR, (pre-)XDR |  |  |  |  | 0.25 | 0.17 | 0.31 | 0.17 | 0.09 | 0.26 | (16)** |
| *the probability of default is assumed to be a function of the regimen, and not of the resistance pattern.  **Adjusted for exclusion of non-report, so the sum of treatment success, failure, died and defaulted add up to 100%  †Outcome probabilities are weighted assuming 93% new and 7% previously treated patients  MDR=multi-drug resistance, defined as resistance to rifampicin and isoniazid (22).  XDR=extensively drug-resistant  FQ=fluoroquinolones  INHmono=isoniazide mono-resistance  PDR=first-line polydrug resistance but not including rifampicin  SLID=injectable aminoglycosides | | | | | | | | | | | | |
